# As above, not so below: Ion fractionation in planetary analog ices

**DOI:** 10.1126/sciadv.ady6763

**Published:** 2026-04-08

**Authors:** Jacob J. Buffo, Mark G. Fox-Powell, Andrii Murdza, Tara C. Tomlinson, Alexa Schultz, Timothy Barton, Caroline Gurd, Angus McEwen, Natalie S. Wolfenbarger, Chase J. Chivers, Britney E. Schmidt, Colin R. Meyer

**Affiliations:** ^1^ Dartmouth College, Hanover, NH, USA.; ^2^ AstrobiologyOU, Faculty of Science, Technology, Engineering, and Mathematics, The Open University, Milton Keynes, UK.; ^3^ School of Environment, Earth, and Ecosystem Sciences, The Open University, Milton Keynes, UK.; ^4^ Los Alamos National Laboratory, Los Alamos, NM, USA.; ^5^ Cornell University, Ithaca, NY, USA.

## Abstract

The geophysical evolution and astrobiological potential of ocean worlds are indelibly linked to the chemical compositions of their oceans and ice shells. In the absence of direct measurements, empirical estimates of subsurface ocean compositions have relied on the assumption that the ionic compositions of ice shell surfaces are representative of their underlying ocean compositions. Here, we present experimental results demonstrating that ion fractionation—the differential entrainment of ion species in forming ices—is likely a prevalent process on ocean worlds, suggesting that planetary ice shell compositions do not directly reflect their underlying ocean compositions. We measure in-ice depletions and amplifications of relative ion concentrations ranging between −40 and +77%, compared to the parent fluid composition. Although this may complicate the interpretation of spacecraft data, ion fractionation provides a mechanism for generating compositionally diverse ices that could help explain the geological complexity of planetary ice shells.

## Teaser

Laboratory experiments show how the physics of salty ice could change our understanding of ocean worlds across the solar system.

## INTRODUCTION

### Background

There is a growing consensus that ocean-derived salts play a key role in the geophysical evolution and habitability of ice-ocean worlds (*1*, *2*). This is bolstered by several observations, including (i) the association of endogenic salt deposits with distinct geomorphological features on icy moon surfaces (*3*–*5*); (ii) the ability of salts to depress the freezing temperature, and thereby extend the longevity, of liquids within planetary ice shells (*6*, *7*); (iii) the critical role salts play in governing the material properties, biogeochemistry, and habitability of salt-rich ices and brines on Earth (*8*–*11*); and (iv) the fundamental role small melt fractions and impurity levels play in governing the geophysics of the analogous terrestrial core-mantle-lithosphere system (*2*, *12*, *13*). Moreover, as direct products of their progenitor oceans, planetary ice shells—and the impurities they contain—constitute observable and accessible geochemical fingerprints of subsurface ocean properties (*2*, *14*, *15*). This is particularly advantageous given the technological challenges of directly sampling the interior oceans of icy worlds (*16*) that cannot sustain persistent direct ocean-to-surface connection via fractures/plumes (e.g., Europa) (*17*–*19*). Accordingly, constraining the composition, concentration, and distribution of salts within planetary ice shells has blossomed into an active field of study and constitutes a primary science objective for the upcoming Europa Clipper and Jupiter Icy Moons Explorer (Juice) missions (*20*, *21*).

To date, quantitative studies of ocean worlds, both observational and theoretical, have presupposed that when salts are entrained in planetary ice shells, the ionic composition (i.e., relative ion abundances) of the resultant ice is directly representative of the ion ratios in the parent fluid. That is, while the total ion content (i.e., bulk salinity) of ice shells is expected to vary depending on how fast the ice formed and how saline the parent fluid is, all dissolved ions have been assumed to be entrained into the ice at the same rate (*14*, *15*). While studies of the equilibrium and fractional crystallization pathways of planetary brines undergoing freezing demonstrate their complex precipitate-driven geochemical evolution (*3*, *22*–*24*), these results exclude multiphase fluid dynamics (assuming complete rejection or retention of salts in the ice phase), either decoupling or completely coupling ice and brine chemistry. As such, we have a modern conceptual ethos of planetary ice shells in which heterogeneities in ice shell ionic composition reflect proportional variations in the chemistry of the source water.

Recent research (*25*, *26*), however, suggests that ion fractionation during freezing—the preferential entrainment/rejection of ion species into/out of forming saline ices at different rates due to coupled geochemical and reactive transport processes (*27*)—could be prevalent under a wide range of ice-ocean world thermodynamic conditions. If this is the case, the ionic speciation within planetary ices may not be directly representative of the progenitor fluid reservoirs from whence they came (*25*). This would substantially complicate the use of ice shell or surface geochemistry as a means of inferring subsurface ocean or brine reservoir compositions, warranting reassessment of ocean composition estimates that have implemented such a method (*4*, *28*, *29*). Moreover, the complex multiphase advection-reaction-diffusion dynamics associated with ion fractionation could provide multiple mechanisms for introducing a diverse range of geochemical and associated material property heterogeneities (e.g., melting point, density, mechanical strength, rheology, etc.) into the ice shells of ocean worlds (*14*). Geochemical variations would directly affect the spatiotemporal habitability of ice shells by governing interstitial brine volumes and properties such as water activity, ionic strength, pH, and chaotropicity/kosmotropicity (*8*, *30*), while physicochemical heterogeneities are putative drivers of several potential ice shell geophysical processes (*1*) (e.g., solid-state convection and eutectic melting) that are hard to generate/reconcile with contemporary models of brine-freezing pathways (*31*) and salt entrainment, which suggest a tendency toward the formation of chemically homogeneous, low-salinity or salt-free ice as ice shells thicken (*14*, *15*, *20*, *22*, *32*, *33*). Accordingly, estimates of ice-ocean world composition, dynamics, and habitability have the potential to be substantially improved by accounting for the combined geochemical and reactive transport physics occurring within porous saline ices (*1*).

Equivalent multiphase processes are known to drive chemical fractionation and property variation patterns in solidifying magmatic and metallurgic systems (*34*–*37*), as well as in sea ice and hypersaline lake ices on Earth (*25*, *27*, *38*, *39*). Natural saline ice samples are subject to complex thermophysical and geochemical processes (e.g., freeze-thaw cycles, meltwater flushing, and reactive transport), the cumulative result of which governs their composition (*40*–*43*). A range of positive and negative ion fractionation signals has been observed in sea ice, brackish ice, marine ice, and hypersaline lake ice (*25*, *27*, *38*, *39*), attributable to varied ice formation histories, differential ion mobility within the ice microstructure, and the formation of solid salt species (*27*, *31*, *38*, *44*). The variability and complexity of such natural systems, however, have made it challenging to constrain quantitative relationships between ion fractionation magnitudes/trends and the properties of the ice’s formation environment, and the top-down growth of physically realistic and appreciably thick analog ices in controlled laboratory environments requires both substantial cold-room facility resources and time (*45*). Furthermore, there exists only a handful of studies (*8*, *39*) investigating the geochemistry of columnar ice formed from brines with diverse (nonseawater) ionic compositions that may be more relevant to ocean worlds [e.g., magnesium sulfate (MgSO_4_)–dominated (*3*, *46*–*48*) and sodium bicarbonate (NaHCO_3_)–dominated systems (*49*–*52*)].

Here, we describe a set of top-down solidification experiments that demonstrate the occurrence of distinct and complex ionic fractionation patterns in compositionally diverse planetary analog ices grown from Earth and notional Europa and Enceladus ocean compositions [sodium chloride (NaCl)–dominated, MgSO_4_-dominated, and NaHCO_3_-dominated], including depletions and amplifications of major ion species relative concentrations of up to −40 and +77%, respectively, compared to the parent fluid compositions. We show that both multispecies ion diffusion and salt precipitation within the forming ice layer play a critical role in governing the ionic composition of the resultant ice and that the final mineral assemblage of the system is affected by several factors, including the rate of ice formation, the ionic composition of the parent brine, and the concentration of the parent brine, consistent with previous studies of both natural and laboratory-grown saline ices (*27*, *31*, *40*, *44*, *53*–*55*). Coupled with the physics of multiphase reactive transport [e.g., (*2*)], ion fractionation presents us with an expanded picture of cryopetrological (pertaining to the origin, structure, mineralogy, and composition of ices) complexity that is likely ubiquitous among the ice shells of ocean worlds. We discuss how ion fractionation can provide a mechanism for generating geochemical heterogeneities in planetary ice shells and the implications this has for the geophysics and habitability of ice-ocean worlds as well as our ability to infer subsurface ocean properties using observations of their overlying ice. Consequently, we highlight both the importance of constraining the extensive multivariate (i.e., thermal, physical, and chemical) parameter space that governs the diversity of planetary ice compositions that can be generated by ion fractionation dynamics and underscore key priorities for future investigations.

### Ion fractionation in saline ices

Ion fractionation in ocean- and brine-derived ices, defined as the deviation in their relative ion concentrations compared to the compositional ratios of their parent fluids, is driven by three physical mechanisms: (i) variable molecular diffusion rates of different solutes in multicomponent aqueous solutions; (ii) precipitation of solid salts; and (iii) differential incorporation of ions into the solid ice crystalline lattice (*27*, *38*, *56*, *57*). Throughout this work, we adopt the conventional quantitative definition of ion fractionation (*27*, *38*)

$${∆}_{X/{\text{Cl}}^{-}}=\frac{\frac{C_{X}}{C_{{\text{Cl}}^{-}}}-{\left(\frac{C_{X}}{C_{{\text{Cl}}^{-}}}\right)}_{\text{initial}}}{{\left(\frac{C_{X}}{C_{{\text{Cl}}^{-}}}\right)}_{\text{initial}}}$$ (1)

which normalizes the system using chloride concentrations due to its typically high solubility and late-stage participation in precipitation reactions. ${∆}_{X/{\text{Cl}}^{-}}$ is the fractionation of ion X (e.g., Mg^2+^, K^+^, SO_4_^2−^, and Na^+^) in the ice sample of interest, $C_{X}$ is the concentration [in parts per thousand (ppt) wt %] of ion X, $C_{{\text{Cl}}^{-}}$ is the concentration of chloride (in ppt wt %), and the initial subscript indicates concentration values of the brine from which the ice formed.

If all the ionic species present within a brine diffused at the same rate, were able to remain in solution until the ice reached its percolation threshold (the point at which interstitial brine becomes hydraulically disconnected from the adjacent fluid reservoir), and were accommodated in the solid ice crystal lattice as clathrates/defects equally, the resultant ionic composition of the ice would mirror that of the fluid from which it formed [e.g., (*6*)]. Natural brines, however, are often not so well behaved.

Dissolved ion species inherently have different molecular diffusivities, and although Coulombic interactions between oppositely charged ions in simple binary electrolytes (e.g., NaCl) can couple dispersion rates [e.g., (*58*)], the ion-ion cross-coupling of more complex (>2 species) multicomponent electrolyte solutions leads to nonuniform ion diffusion in porous media (*58*), of which saline ices are a prime example (*41*). In ocean-derived ices formed via top-down solidification (e.g., sea ice and planetary ice shells), a strong interstitial brine concentration gradient will drive ion diffusion toward the underlying fresher ocean (*14*, *15*, *42*). The exact diffusion rates of each ion species depend on both its self-diffusivity and concentration gradient as well as the concentration gradients and diffusivities of all the other anions and cations in the solution [e.g., (*58*)], but, generally, high-mobility ions (e.g., K+) are expected to outpace low-mobility ions (e.g., SO_4_^2−^ and Mg^2+^), leading to negative and positive Cl^−^-normalized fractionation signals within the resulting ice, respectively (*25*, *27*).

In addition to differential diffusion rates in the permeable regions of saline ices, the low temperatures and high salinities of their interstitial brines constitute environmental conditions with a high propensity for solid salt precipitation. If thermochemical solubility limits are exceeded [e.g., approximately −6.3°C for mirabilite (Na_2_SO_4_·10H_2_O) formation in the seawater-freezing pathway (*31*, *59*)], solid salt species will begin to form within the ice (*59*, *60*). The formation of this additional phase will, in turn, alter the chemical composition, density, freezing point, and dynamics of the residual brine (*61*). As freezing continues and dissolved ion species are diffused or convected out of the system, it is assumed that the precipitated solid salt species will not be removed from the system (*27*). This is consistent with the retention of precipitates in other hydrogeological systems [e.g., mineral precipitation and cementation in aquifers; (*62*, *63*)]. The result is positive fractionation signatures for the constituent ion species of the precipitates (*27*, *38*).

The final mechanism that can facilitate ionic fractionation in saline ices—differential ion incorporation into the solid ice crystalline lattice—becomes important at very slow growth rates. While the overwhelming majority of ions are excluded from the ice crystalline lattice, a small, but nonzero, amount of ions is entrapped within the growing crystals, either within hexagonal cavities or as replacement ions in the crystal structure (*64*). While the partition coefficients (k) governing the rates of ion incorporation into the solid ice phase are such that ion entrainment in columnar ice via pore close-off of brine pockets/channels will occur at orders-of-magnitude higher levels, rendering the solid-phase partitioning signals negligible, there exists a critical ice-ocean interface growth velocity [estimated to be <10^−9^ to 10^−12^ m/s for Earth’s ocean; (*65*, *66*)] where the stability of the interface shifts from cellular/lamellar to planar, inhibiting the formation of brine pockets/channels and leaving lattice incorporation of ions as the only mechanisms for salt entrainment in the ice. The value of this critical velocity is not well constrained and depends on multiple factors such as ocean concentration, differential ion partition coefficients, and ocean thermal gradients (*25*). While this regime may be important for the ice-ocean interfaces of thick planetary ice shells (*25*), it is characterized by growth rates far lower than the minimal growth velocities attainable in laboratory experiments and even those observed at the base of terrestrial ice shelves [e.g., (*65*), wherein a columnar ice structure in sea ice growing at 3.17 to 4.12 × 10^−10^ m/s, or 1 to 1.3 cm/year was observed). Given the experimental nature of the current work, we neglect the effects of lattice incorporation in the analysis of our results, but constraining the critical growth velocities of thermocompositionally diverse ice-brine systems (e.g., via the extrapolation of critical growth velocity curves for dilute systems, which are easier to obtain in laboratory experiments given their larger values) is an important area of research that is directly relevant to understanding the geochemistry and geophysics of deep ice-ocean environments. Below these critical growth velocities, it is expected that ion fractionation will be driven by variations in ion partition coefficients (i.e., for ion X, $k_{X}>k_{{\text{Cl}}^{-}}$ would result in positive fractionation) (*25*). The cumulative and coupled effects of multispecies ionic diffusion, solid salt precipitation, ion accommodation in the ice crystal lattice, and multiphase reactive transport will govern the ionic composition of the resultant ice.

## METHODS

### Ice fabrication and processing

Eight planetary analog ice samples were grown via top-down solidification in Dartmouth’s Ice Research Laboratory. Three different initial brine compositions were selected to represent the hypothesized ocean chemistries of Europa and Enceladus: (i) a NaCl-dominated ocean similar to that of Earth’s (*4*); (ii) a MgSO_4_-dominated ocean (*3*, *46*); and (iii) a NaHCO_3_-dominated ocean (*51*, *66*). In addition, various initial brine concentrations were implemented to investigate the effect of ocean salinity on the resultant ice chemistry and fractionation. The initial brine ionic compositions and concentrations for all eight experiments can be seen in Table 1. We note that other ocean composition estimates exist for these icy worlds [e.g., the Na─SO_4_─C–dominated ocean chemistry predicted for Europa in (*67*)], which could be explored in future experiments.

**Table 1.** Brine geochemistry and experiment duration. Initial brine ionic compositions and TDS in grams per kilogram weight percent (parts per thousand), as measured by the IC and ICP-OES techniques described in the “Chemical analysis” section in Methods. Also included is the duration of each experiment, in days.

Experiment name	Ca^2+^ (ppt)	Cl^−^ (ppt)	HCO_3_^−^ (ppt)	K^+^ (ppt)	Mg^2+^ (ppt)	Na^+^ (ppt)	SO_4_^2−^ (ppt)	TDS (ppt)	Duration (days)
Seawater 1	0.33	13.53	-	0.35	1.00	4.74	1.75	21.71	7.82
Seawater 2	0.36	13.57	-	0.41	1.09	4.17	1.75	21.34	20.85
Europa 1	0.36	0.64	-	0.10	1.51	0.68	7.36	10.66	8.82
Europa 2	0.34	0.61	-	0.09	1.23	0.56	5.86	8.68	24.81
Europa 3	0.37	1.76	-	0.45	5.41	2.23	27.01	37.22	46.93
Enceladus 1	-	1.15	0.87	0.09	-	1.03	-	3.14	16.86
Enceladus 2	-	25.28	17.25	2.57	-	28.10	-	73.20	22.83
Enceladus 3	-	8.00	5.93	0.72	-	8.62	-	23.26	40.88

**Fig. 1.**
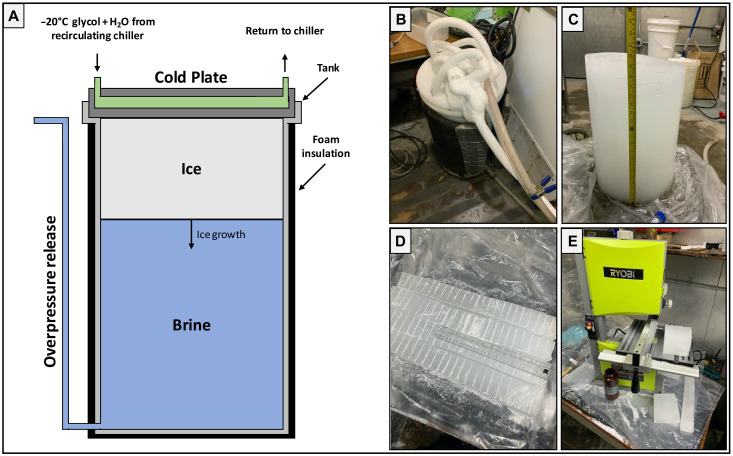
Growth and processing of planetary analog ice samples at the Dartmouth’s Ice Research Laboratory. (**A**) Schematic of the ice growth apparatus. (**B**) Image of the ice growth apparatus shortly after the onset of an experiment. (**C**) The ~50-cm-thick Europa 2 ice column immediately after extraction. The column is placed upside down to reduce brine loss from the highly porous basal region of the ice. (**D**) Ice columns subsectioned into smaller, more easily workable pieces and scored at 2-cm intervals in preparation for vertical partitioning. (**E**) The scored subsections in (D) were cut using an electric band saw, and the 2-cm layers were placed into 500-ml high-density polyethylene amber Nalgene sample bottles. These samples were subsequently melted, filtered, and processed via IC, ICP-OES, and NDIR spectroscopy to acquire our ionic composition and ionic fractionation profiles.

To begin an experiment, we completely dissolved laboratory-grade salts in a 57-liter linear low-density polyethylene sidewall insulated (5-cm low-density polyurethane) tank using local tap water. The fluid-filled portion of the tank is 65.5 cm deep and 35 cm in diameter. The tank was fitted with a free-flowing pressure release hose at the base to prevent overpressurization of the brine during top-down freezing (Fig. 1, A and B). The tank was placed in a +2°C cold room, covered, and left to thermally equilibrate. Once equilibrated, an initial brine composition sample was taken (Table 1), the surface of the brine was seeded with ~1 cm of laboratory-produced freshwater snow (produced by freezing tap water and milling the resulting ice block into fine swarf) to ensure laterally homogeneous nucleation sites for the onset of crystallization, and a glycol-chilled aluminum cold plate was placed in contact with the surface of the brine on top of the tank (Fig. 1, A and B). Waterproof temperature data loggers (Onset HOBO 64K Pendant) were placed on the cold plate and in the brine to record the top and bottom temperature of the growing ice column throughout the duration of the experiment (section S1). In all experiments, the driving glycol chiller temperature was set to −20°C, and top-down freezing was left to progress for 8 to 47 days.

At the end of an experiment, the glycol plate was warmed and removed, the remaining underlying brine was drained via the pressure release hose, and a “final brine” sample was taken for compositional analysis. The sides of the tank were mildly warmed using a heat gun until the entire ice column could be removed, ensuring that no thermal cracks were created (Fig. 1C). The ice was immediately placed upside down in a plastic bag, to limit brine drainage from the lower porous portions of the ice, and put in a −30°C cold room to freeze the residual brine in place and maintain the compositional profile of the ice at the time of its extraction.

To acquire ionic composition profiles of the ice, the ice columns were cut into smaller sections in a −10°C cold room, and 2-cm vertical increments were measured throughout the depth of the ice column (Fig. 1D). The 2-cm layers were partitioned using a band saw, and ice from each layer was collected in 500-ml amber Nalgene bottles (Fig. 1E). The ice was then melted by placing the Nalgene bottles in a hot water bath. To prepare the resultant melt samples for chemical analysis, we filtered them using 0.22-μm polyethersulfone filters to remove any particulates.

### Chemical analysis

Elemental and ionic concentrations in all melted ice and brine samples were quantified in the Ecosystems Laboratories at The Open University. Na^+^, K^+^, Mg^2+^, and Ca^2+^ were quantified via inductively coupled plasma optical emission spectroscopy (ICP-OES) using an Agilent 5110. Liquid samples were diluted to factors between 10 and 1000× in ultrapure 2% nitric acid prior to analysis. Wavelengths used for element quantification were (in nanometers): Na^+^, 589.592; K^+^, 766.491; Mg^2+^, 285.213; and Ca^2+^, 317.933. Chloride and sulfate were quantified via ion chromatography (IC) using a Metrohm 930 Compact IC Flex. Samples were diluted in ultrapure H_2_O prior to analysis. Triplicated sample measurements were included every five to 10 samples; standard deviation of triplicates was <0.2% in all cases, conservatively propagating to ion fractionation errors of <0.71% for all measurements (Eq. 1). Dissolved inorganic carbon was first converted to CO_2_ and subsequently quantified through nondispersive infrared (NDIR) spectroscopy using an Elementar Vario TOC Cube. Samples were diluted in ultrapure H_2_O prior to analysis. For all analytical techniques, potential contributions of sample matrix to chemical data were corrected for by measuring “blank” samples of ultrapure H_2_O or nitric acid (as appropriate), and drift monitoring was conducted through repeat measuring of internal standards within each run.

### Geochemical modeling

We performed complementary geochemical modeling of the ice columns to characterize the predicted distribution and speciation of precipitated salts and investigate the consistency with regions that exhibit positive fractionation signals. We used the open-source equilibrium geochemistry software PHREEQC (*68*) and its frezchem.dat database (*69*, *70*) to simulate the mineral assemblages present in each vertical sample at the time of extraction. As inputs for each sample, we use the ionic composition results of the IC, ICP-OES, and NDIR analysis and assume a linear conductive thermal profile within the growing ice, bounded by the measured cold plate temperature at 0-cm depth and the measured brine temperature at the ice-brine interface. This assumption is supported by previous measurements of natural and laboratory-grown sea ice (*71*, *72*) as well as thermal data from experiments where temperature sensors were frozen into the growing ice (see section S2). No restrictions were placed on the kinetics or speciation of the precipitation reactions available to PHREEQC during the simulations.

## RESULTS

### Ionic composition

**Fig. 2.**
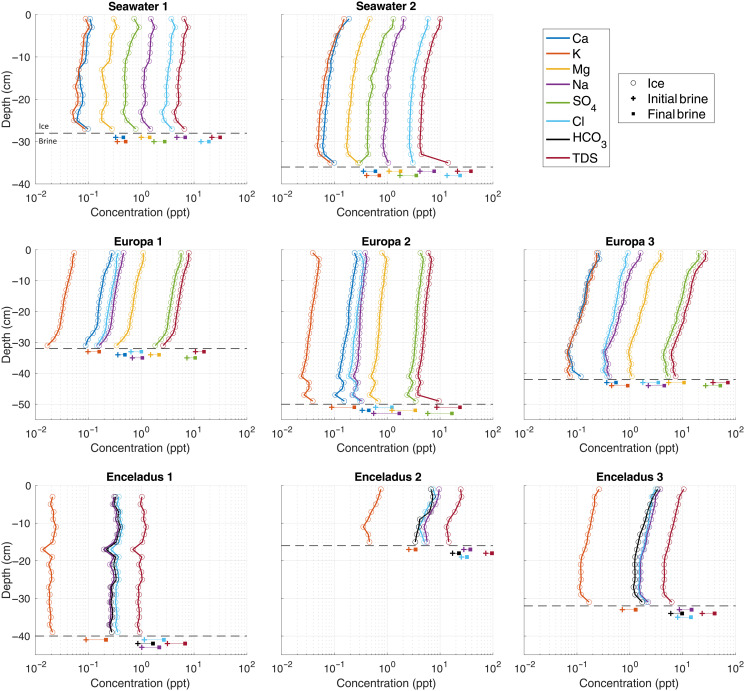
Ion composition profiles of laboratory-grown planetary analog ices and their underlying brines. Decreasing ion concentrations with depth, observed in all ice columns, are consistent with increased brine drainage efficiency as the ice thickens and thermal gradients (ice growth rates) at the ice-brine interface decrease. Slight increases in ion concentrations near the base of the ice columns are associated with the high porosity, active, multiphase (mushy) layer and are consistent with previous measurements of sea ice salinity profiles [e.g., (*110*–*112*)]. The horizontal dashed black line indicates the thickness of the ice column at the time of extraction. The measurements of brine ionic composition (both initial and final) are vertically offset for ease of visualization and are representative of the entire well-mixed underlying brine column (*43*). Cryoconcentration of ions in the underlying brine as the experiments evolve is a hallmark of confined freezing systems and provides an analog to putative perched/isolated water systems in planetary ice shells. Note that depth scales are consistent across each row, and measurement errors are within the symbol size for all values.

The ionic composition profiles of the eight ice columns grown during our experiments can be seen in Fig. 2. They display the mass weight percent [g (solute)/kg (solution)≡parts per thousand≡ppt] of the major ions present throughout the ice as well as in the initial brine and final underlying brine (see data S1 for all values reported throughout the text). Also included is the mass weight percent of the total dissolved solids (TDS) in the system, which is equivalent to bulk salinity. Nearly all the ice columns exhibit characteristic “c-shaped” profiles, akin to those observed in natural and laboratory-grown sea ice, although they appear muted in log-space. The two exceptions are the Europa 1 profile, which has its lowest ion and TDS values at the base of the ice column, likely due to interstitial brine loss from the porous basal region of the ice during extraction; and the Enceladus 1 profile, which does not exhibit significant variability in ionic composition or TDS with depth, potentially due to the low salinity of the initial brine, preventing strong/distinguishable signatures given the lower concentrations of all species. The general decrease in ion concentration with depth is caused by the increasing efficiency of brine drainage as the ice thickens and the thermal gradients within the active basal portion of the ice decrease. Cryoconcentration of all ions in the final brines occurs due to the brine rejection from the forming ice and the finite nature of the tank used in the experiment, sometimes to the point of saturating the underlying brine and causing precipitation of salts at the base of the tank (e.g., fig. S3).

### Ionic fractionation

**Fig. 3.**
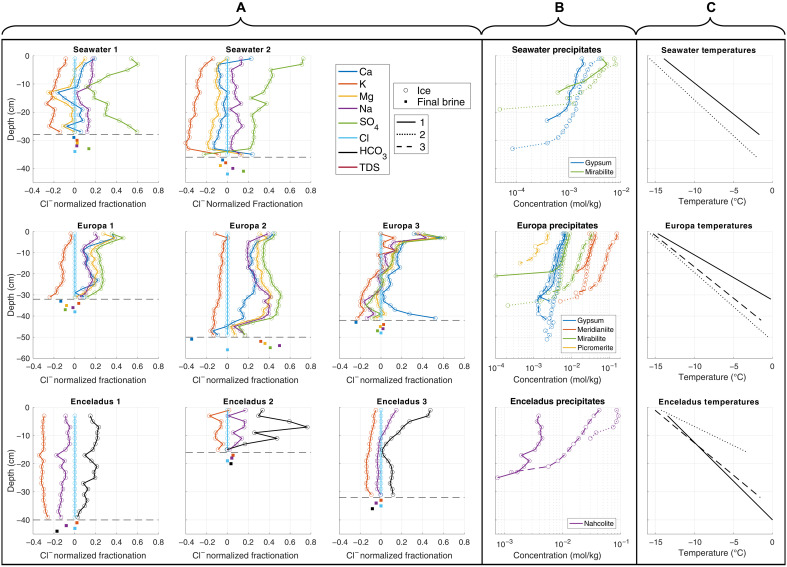
Ion fractionation profiles. (**A**) Chloride-normalized ion fractionation profiles in the ice columns and final underlying brines of the experiments described in Table 1. A diverse and complex array of fractionation signals are evident across the different ice columns. Seawater ice columns exhibit positive Na^+^ and SO_4_^2−^ fractionation and negative K^+^ fractionation. The Europa 1 and 2 ice columns exhibit strong Na^+^, Mg^2+^, Ca^2+^, and SO_4_^2−^ fractionation and negative K^+^ fractionation. The Europa 3 ice column exhibits muted K fractionation in the upper layers of the ice column and no Na^+^, Mg^2+^, Ca^2+^, and SO_4_^2−^ fractionation in the lower portions of the ice column. All Enceladus ice columns exhibit negative K^+^ fractionation and positive to negligible Na^+^ and HCO_3_^−^ fractionation, depending on the starting brine concentration and depth within the ice column. (**B**) Simulated precipitate distribution in the ice columns in (A), generated using PHREEQC, the ionic compositions of Fig. 2, and the interpolated final ice column temperature profiles shown in (C). Different ice columns are represented by different line styles (column 1, solid; column 2, dotted; column 3, dashed). Ions associated with the predicted mineral assemblages [gypsum, CaSO_4_·2H_2_O; mirabilite, Na_2_SO_4_·10H_2_O; meridianiite, MgSO_4_·11H_2_O; picromerite, K_2_Mg(SO_4_)_2_·6H_2_O; nahcolite, NaHCO_3_] consistently exhibit corresponding positive fractionation signals in the empirical data, suggesting that the dynamics of solid salt precipitation in saline ices can play a distinct role in governing their composition. (**C**) The vertical temperature profiles in the ice columns at the time of their extraction, linearly interpolated from the measured cold plate and underlying brine temperatures (see sections S1 and S2). Note that depth scales are consistent across each row, and measurement errors are within the symbol size for all values.

The chloride-normalized ion fractionation profiles of the eight ice columns analyzed during this study, as well as the fractionation of their final underlying brines, can be seen in Fig. 3A. Every ice column analyzed during this study exhibited significant positive and negative ion fractionation signals (Table 2) consistent with differential multispecies diffusion and salt precipitation processes. However, the significant variability in fractionation levels (−40 to +77%) and distributions between ice columns grown from brines of different compositions and/or concentrations suggests that a complex interplay of reactive physicochemical processes are operating within these multiphase ice-brine systems.

**Table 2.** Ice column mean fractionation trends. All ice columns exhibit negative K^+^ fractionation. Seawater-derived ice columns exhibit positive Na^+^ and SO_4_^2−^ fractionation. Europa ocean analog–derived ice columns exhibit positive Ca^2+^, Mg^2+^, Na^+^, and SO_4_^2−^ fractionation. Enceladus ocean analog–derived ice columns exhibit positive HCO_3_^−^ and Na^+^ fractionation. We report standard deviations (σ) and standard errors of the means, calculated as $\sigma /\sqrt{n}$ (*113*). As we expect depth-dependent variance in fractionation signals, due to evolving multiphase dynamics and environmental pressures, we stress the utility of standard deviation as a metric for assessing the significance/strength of a fractionation signal rather than its uncertainty.

Experiment (no. of samples)	${\bar{\Delta }}_{Ca^{2+}/{\text{Cl}}^{-}}$ (σ)	${\bar{∆}}_{{\text{HCO}}_{3}^{-}/{\text{Cl}}^{-}}$(σ)	${\bar{∆}}_{K^{+}/{\text{Cl}}^{-}}$ (σ)	${\bar{∆}}_{{\text{Mg}}^{2+}/{\text{Cl}}^{-}}$ (σ)	${\bar{∆}}_{{\text{Na}}^{+}/{\text{Cl}}^{-}}$ (σ)	${\bar{∆}}_{{\text{SO}}_{4}^{2-}/{\text{Cl}}^{-}}$ (σ)
Seawater 1 (*n* = 14)	0.025 ± 0.024 (0.089)	-	−0.184 ± 0.014 (0.053)	−0.045 ± 0.024 (0.091)	0.121 ± 0.013 (0.050)	0.365 ± 0.044 (0.164)
Seawater 2 (*n* = 18)	−0.032 ± 0.024 (0.103)	-	−0.292 ± 0.020 (0.083)	−0.100 ± 0.017 (0.074)	0.081 ± 0.009 (0.040)	0.323 ± 0.048 (0.203)
Europa 1 (*n* = 16)	0.159 ± 0.025 (0.101)	-	−0.115 ± 0.013 (0.053)	0.190 ± 0.021 (0.083)	0.128 ± 0.011 (0.045)	0.261 ± 0.019 (0.076)
Europa 2 (*n* = 25)	0.191 ± 0.026 (0.131)	-	−0.083 ± 0.008 (0.040)	0.293 ± 0.021 (0.106)	0.266 ± 0.019 (0.097)	0.379 ± 0.022 (0.112)
Europa 3 (*n* = 21)	0.173 ± 0.032 (0.146)	-	−0.071 ± 0.024 (0.109)	0.081 ± 0.030 (0.140)	0.026 ± 0.033 (0.150)	0.070 ± 0.039 (0.178)
Enceladus 1 (*n* = 20)	-	0.145 ± 0.014 (0.061)	−0.301 ± 0.004 (0.017)	-	0.106 ± 0.008 (0.037)	-
Enceladus 2 (*n* = 8)	-	0.361 ± 0.086 (0.244)	−0.080 ± 0.020 (0.056)	-	0.100 ± 0.024 (0.068)	-
Enceladus 3 (*n* = 16)	-	0.153 ± 0.034 (0.137)	−0.106 ± 0.008 (0.030)	-	0.009 ± 0.015 (0.058)	-

Figure 3B depicts the PHREEQC-simulated precipitate distributions within the ice columns using their measured ionic compositions and the linearly interpolated conductive temperature profiles within the ice (Fig. 3C). These precipitate distributions provide a benchmark to aid in the interpretation of the experimental fractionation data and can be used to identify where the empirical systems deviate from the geochemical equilibrium conditions imposed by PHREEQC. A summary of the salts expected to form in each experiment and the temperature regimes they occupy can be found in Table 3. We note that the minimum ice temperatures reached in our experiments are on the order of −15°C and that additional speciation may continue upon further cooling if other salts become more thermodynamically favorable [e.g., (*22*, *31*)].

**Table 3.** In situ precipitate stability. Salt occurrence and thermal stability within the laboratory-grown ice columns, as predicted by PHREEQC.

Experiment	Precipitate	Observed temperature range (°C)
Seawater 1	Gypsum	−13.9 to −3.9
	Mirabilite	−13.9 to −8.5
Seawater 2	Gypsum	−15.7 to −3.2
	Mirabilite	−15.7 to −8.7
Europa 1	Gypsum	−14.7 to −0.2
	Mirabilite	−14.7 to −5.3
	Meridianiite	−14.7 to −6.3
Europa 2	Gypsum	−15.6 to −0.6
	Mirabilite	−15.6 to −5.2
	Meridianiite	−15.6 to −5.8
Europa 3	Gypsum	−15.3 to −1.5
	Mirabilite	−15.3 to −5.8
	Meridianiite	−15.3 to −5.8
	Picromerite	−15.3 to −10.6
Enceladus 1	Nahcolite	−13.4 to −5.4
Enceladus 2	Nahcolite	−14.2 to −6.9
Enceladus 3	Nahcolite	−15.0 to −5.5

Both seawater ice columns exhibit positive Na^+^ and SO_4_^2−^ fractionation signals, indicative of the precipitation of a Na_2_SO_4_-bearing mineral. This is consistent with the precipitation of mirabilite (Na_2_SO_4_·10H_2_O), as predicted by PHREEQC, in the upper portions of the ice columns. Both ice columns also exhibit negative K^+^ fractionation, which is consistent with the amplified diffusivity of potassium ions in multispecies electrolyte solutions (*58*). This negative trend increases with depth as ice growth slows and there is more time for the ions to diffuse out of the lower permeable regions of the ice. Differential ion diffusivity can also explain the persistent positive fractionation of Na^+^ and SO_4_^2−^ in the lower portions of the ice column where mirabilite is not expected to form, as SO_4_^2−^ has a reduced diffusivity compared to Cl^−^ and Na^+^ will tend to charge couple to SO_4_^2−^. While gypsum (CaSO_4_·2H_2_O) is predicted to precipitate in the PHREEQC simulation, there are no strong signals of positive Ca^2+^ fractionation (<1σ; Table 2). There are two potential causes of this discrepancy. Either the lower levels of gypsum expected to form (compared to mirabilite) are not enough to cause a significant fractionation signal, or, more likely, there is an inhibition of gypsum formation occurring within the ice. Gypsum precipitation is a sluggish reaction at 25°C (*73*, *74*) and will be hindered further at subzero temperatures (*31*). Moreover, high-salinity environments, such as the interstitial brines considered here, are known to increase the solubility and decrease the nucleation and growth rates of gypsum (*75*), providing kinetic inhibitions to the accumulation of gypsum within the ice. These inhibition processes would increase the concentrations of dissolved SO_4_^2−^ in the ice, providing a reservoir and geochemical pathway for the formation of additional mirabilite in the lower reaches of the ice, consistent with the positive fractionation signals observed in the basal regions of both columns. If these systems were able to truly equilibrate, as simulated by PHREEQC (Fig. 3B), the expectation is that mirabilite dissolution at low temperatures would provide the sulfate needed for gypsum formation (*31*). However, continued freezing could limit interstitial brine contact with entrained mirabilite crystals, and prior studies (*31*) have demonstrated that even under ice-free conditions, the equilibration of mirabilite to gypsum at subzero temperatures is exceptionally slow (disequilibrium persisting after 12 weeks at −26°C).

All the Europa ice columns exhibit positive Ca^2+^, Mg^2+^, Na^+^, and SO_4_^2−^ fractionation signals, indicating the precipitation of minerals bearing these species. This is consistent with the precipitation of meridianiite (MgSO_4_·11H_2_O), mirabilite (Na_2_SO_4_·10H_2_O), and gypsum (CaSO_4_·2H_2_O), as predicted by PHREEQC. The ice columns from the Europa 1 and Europa 2 experiments exhibit negative K^+^ fractionation across all depths, while the ice column from the Europa 3 experiment exhibits negative K^+^ fractionation below ~17-cm depth and a negligible to slightly positive K^+^ fractionation signal above ~17 cm. Again, negative K^+^ fractionation can be attributed to the amplified diffusivity of potassium, while the spatial extent of the negligible to positive K^+^ fractionation signal of the Europa 3 ice column corresponds exceptionally well to the 1- to 15-cm region predicted to form the K-bearing double-salt picromerite [K_2_Mg(SO_4_)_2_·6H_2_O]. Both experimental and theoretical works have shown that as MgSO_4_ molality increases, the K_2_SO_4_ molality needed to precipitate picromerite decreases [see figure 13 in (*76*)], which could explain the presence of picromerite in the more concentrated Europa 3 experiment and the absence of a picromerite signal in the more dilute Europa 1 and Europa 2 experiments. The ability to form gypsum in these experiments is likely due to the amplified concentrations of sulfate and reduced levels of sodium. The Europa solutions will saturate with respect to gypsum at higher temperatures than the Seawater experiments (*3*, *31*) given the reduced freezing point depression capabilities of their primary ions, potentially reducing the thermally driven kinetic inhibition of precipitation. In addition, the lower sodium concentrations may reduce the amount of mirabilite precipitation occurring, reducing a sulfate sink that could have hindered gypsum formation in the Seawater experiments.

There is a distinct difference in the positive fraction trends of the Europa 1 and 2 ice columns and those of the Europa 3 ice column. While the positive fractionation trends in the Europa 1 and 2 ice columns are relatively persistent throughout their entire depths (except for near the base of the columns), the Europa 3 ice column exhibits a more significant decrease in positive fractionation with depth, and by approximately halfway through the ice column (~20 cm), fractionation becomes either negligible or slightly negative. An exception to this is the spike in positive Ca fractionation at the base of the ice column. This variability in fractionation between ice columns can likely be attributed to the difference in initial brine concentration between the experiments. Europa 1 and Europa 2 have initial TDS values of 10.66 and 8.68 ppt, respectively, whereas Europa 3 has an initial TDS value of 37.22 ppt. This variation in salinity fundamentally alters the dynamics of salt precipitation and rejection in the porous basal layer of the ice and the ice structure at the time of these processes. Density (analogous to concentration) gradients between interstitial brines within the ice and the underlying fluid reservoir are needed to drive convection in and desalination of the porous basal ice layer. More complete freezing is needed to drive equivalent concentration gradients in less saline fluids. For example, ice formed from a 10-ppt solution, assuming no salt loss, would need to reach a porosity of ~0.09 to generate an interstitial brine concentration of 110 ppt and a concentration difference of 100 ppt between the interstitial brine and the underlying fluid, while ice formed from a 40-ppt solution would only need to reach a porosity of ~0.29 to generate an interstitial brine concentration of 140 ppt and an equivalent 100-ppt concentration difference. Interstitial brine saturation and salt precipitation events will therefore occur at substantially higher porosities in higher-salinity systems (e.g., Europa 3). If ice permeability is high enough, it is possible this could facilitate the mobility of solid salts, removing or reducing the efficiency of precipitation as a mechanism to drive positive fractionation signals. Meanwhile, these saturation limits will be reached at lower ice porosities in more dilute systems, likely limiting the mobility of any precipitates [e.g., figure 12 in (*77*)] and leading to distinct positive fractionation signals. Ice microstructure, permeability, and brine/salt mobility may be further affected by variations in ice growth rates between the different experiments, where more concentrated brines with lower freezing points drive slower ice column growth leading to larger plate spacing, higher permeabilities, and lower percolation thresholds (*78*). The maintenance of positive fractionation in the upper reaches of the Europa 3 ice column can likely be attributed to the necessity of the ice to reach a critical thickness [i.e., Rayleigh number; (*79*)] before the onset of interstitial brine convection, leading to increased precipitate retention at the top of the ice and the decrease of this signal with depth as convection becomes more efficient and ice growth slows. This same phenomenon causes high salinities at the top of sea ice cores (*79*–*81*). Accordingly, the porosity, permeability, and compositional profiles of these, and other planetary, ices will be dynamic, unique, and heavily dependent on their thermochemical histories.

All the Enceladus ice columns exhibit negative K^+^ fractionation signals consistent with the amplified diffusion rate of K^+^. Positive fractionation signals of Na^+^ and HCO_3_^−^ in nearly all the ice columns are consistent with the precipitation of nahcolite (NaHCO_3_), as predicted by the PHREEQC simulations. The exception is the slightly negative Na fractionation signal in the Enceladus 1 ice column. Given the highly dilute nature of the initial brine used to generate this ice column and the simpler four-component nature of the Enceladus brines, this could be caused by diffusion dominating the system. The higher diffusivity of Na^+^ compared to the larger and less diffusive HCO_3_^−^ ion, along with sodium’s potential propensity to diffusively couple with the oppositely charged chloride ion (*58*), could lead to a negligible Na^+^ fractionation signal and a positive HCO_3_ fractionation signal. This possibility is bolstered by the positive HCO_3_^−^ fractionation in the lower portion of the Enceladus 3 profile while there exists a negligible Na^+^ fractionation signal. This occurs where nahcolite precipitation is simulated to be greatly reduced or nonexistent and, as the only expected precipitate, suggests that diffusion should be the process driving fractionation in this region. Depending on the pH of the interstitial brine, some HCO_3_^−^ loss due to decomposition via HCO_3_^−^ + H^+^ ⇌ H_2_CO_3_ ⇌ CO_2_(aq) + H_2_O may be possible, although we expect these low temperature solutions to be alkaline and for HCO_3_^−^ to be stable in solution. Future investigations would benefit from measuring the pH of both the initial and final underlying brines and the melted ice samples.

Fractionation trends in the final brine samples are less distinct and consistent. Frequently, the magnitude of the underlying brine fractionation trends is muted due to (i) the larger volume of the underlying brine when compared to that of the overlying ice and (ii) the higher ionic concentrations present in the underlying brine (e.g., a difference of 0.5 ppt in relative ion abundance in the low-salinity ice has a larger impact on fractionation than in the higher-salinity brine). The immediate assumption would be that the fractionation trends in the underlying brine at the end of an experiment should be opposite those of the associated ice column, i.e., if there is a depletion of a relative ion abundance in the ice, then conservation of mass suggests there should be an amplification in relative ion abundance in the underlying fluid. There are several situations throughout these experiments where this is the case. Positive K^+^ fractionation is present in the final brines of nearly every experiment, consistent with the negative fractionation signals seen in the overlying ice. Europa 1 and 3 and Enceladus 1 and 3 all exhibit negative fractionation signals in their final brine samples that juxtapose the positive fractionation signals associated with the modeled precipitates in their overlying ices.

Alternately, thicker and higher-salinity ices (e.g., Seawater 1 and 2, Europa 2, and Enceladus 2) exhibit positive ion fractionation trends in their final brine samples that mirror those of the expected precipitates within their overlying ice columns. This is likely due to some amount of precipitated solid salt being present in the final brine samples. This may be caused by the resuspension of precipitates that have formed on the bottom of the tank due to cryoconcentration of the underlying brine (e.g., in the case of Europa 2, see fig. S3) as the tank is being drained, or the presence of fine suspended solid salts in the underlying brine due to their precipitation within the ice column and potential mobilization and rejection due to convective brine drainage. Even if the underlying brine is unsaturated with respect to the rejected salt, the subzero temperature of the liquid could kinetically inhibit or substantially slow the redissolution of these salts (*75*), providing a mechanism for their persistence and possible impact on the resultant final brine measurements. Visual inspection of the final brine samples did not suggest that any solid salts were present; however, these samples were not filtered until after they warmed to room temperature, so redissolution of trace, visually undetectable salts could cause these deviations from expected values/trends. Future studies could reduce these potential contaminations by cold filtering underlying brine samples at or near in situ temperatures immediately upon their extraction.

A distinct limitation of the experiments is the finite nature of the underlying brine reservoir. While the progressive cryoconcentration of the underlying fluid is a relevant simulation of thickening planetary ice shells, which will drive salination of their subsurface oceans [e.g., (*82*)], and solidifying isolated lenses/sills within ice shells [e.g., (*7*, *83*)], this cryoconcentration complicates the definition of fractionation in our laboratory setting as well as the interpretation of the results. Per Eq. 1, fractionation in the ice is measured relative to the ionic concentrations of the brine from whence it formed, which is temporally evolving throughout our experiments. It is unclear, however, at what stage during the ice’s solidification this comparison should be made. Is it at the onset of crystallization at the ice-brine interface? Is it when the ice becomes hydraulically disconnected from the underlying brine? If it is the latter, a comprehensive understanding of the multiphase properties of the ice column and its dependence on composition is needed. Considering this ambiguity, we have elected to calculate all ion fractionations relative to their initial brine compositions at the onset of the experiments (Table 1). As such, our reported fractionation signals constitute conservative lower bound estimates, for we expect Cl^−^ normalized relative ion abundances in the underlying brine [i.e., ${\left(C_{X}/C_{{\text{Cl}}^{-}}\right)}_{\text{initial}}$] to grow increasingly opposed to those in the ice, leading to underestimations. This is consistent with several of our experiments (e.g., Europa 1, Europa 3, Enceladus 1, and Enceladus 3), while several others (e.g., Seawater 1, Seawater 2, and Enceladus 2) exhibit minimal fractionation in their final underlying brines, suggesting limited variation from their initial relative ion abundances. Future experiments could reduce these challenges using a substantially larger tank; however, this poses its own cost and logistical challenges. Presently, this approach to calculating fractionation constitutes the best option for standardizing and comparing our fractionation results between ice columns.

## DISCUSSION

### Implications

At a fundamental level, our results demonstrate that ion fractionation is not only possible but is likely a prevalent process in ices that form via the top-down freezing of saline fluids. In particular, we have shown that the relative ion abundances present in laboratory-grown saline ices can vary significantly from those of their progenitor solutions (−40 to +77%) due to the physics associated with multispecies molecular diffusion in a porous medium and salt precipitation dynamics. Moreover, we show that the magnitudes of these fractionation signals are dependent on the composition and concentration of the parent solution as well as the ice formation rate. Because planetary ice shells likely form and grow in an analogous manner (*15*, *25*), our findings indicate that the geochemistry of planetary ices cannot be assumed to directly represent the composition of their parent fluids. These results have profound implications for the characterization of subsurface oceans and fluid reservoirs on ice-ocean worlds, as the surface geochemical assemblages/abundances that can be observed by spacecraft may not accurately represent the composition, and thus solution properties, of these internal water reservoirs. While contemporary detections of salts on the surfaces of icy worlds [e.g., (*3*, *4*, *28*, *84*)] are useful for identifying likely components of their subsurface oceans, the abundances or dominance of certain salts may be significantly skewed by ion fractionation processes (e.g., amplification of precipitated salts and depletions of ions with high solubility/diffusivity), rendering associated ocean composition estimates inaccurate (*25*). Continued experimental investigations exploring and constraining the relationships between the formation conditions of saline ices and their resultant geochemistry will provide the best opportunity to determine the uniqueness/degeneracy of compositional signatures and establish methods to deconvolve the history and origins of planetary ices using available spacecraft measurements.

From a geological and geophysical perspective, building on the foundational work by sea ice and hypersaline lake ice researchers [e.g., (*27*, *38*, *39*)], ion fractionation presents us with an expanded picture of cryopetrological complexity that is likely ubiquitous among the ice shells of ocean worlds. Our results show that variations in brine composition, brine concentration, and the thermal gradient driving ice formation (i.e., freezing rate) lead to diverse ice salinities, ionic compositions, and mineral assemblages. Given the substantial impact salts have on the thermophysical, mechanical, and phase characteristics of ices [e.g., density (*85*, *86*), viscosity (*87*, *88*), strength (*89*), liquid fraction (*6*), etc.] and the key role material property and phase heterogeneities have been suggested to play in driving geophysical processes capable of producing the diverse geology and geomorphology observed on ocean worlds (*1*), identifying and constraining processes that can facilitate heterogeneous distributions of salt within planetary ices will be instrumental in extending our understanding of these complex and dynamic environments. Critically, the dynamics of ion fractionation have the potential to introduce additional heterogeneities and geochemical complexities, and, by association, variations in material properties, into saline ices, beyond those generated by models of salt entrainment that do not consider ion fractionation [e.g., (*2*, *14*, *15*)].

In addition to material property variations brought about by differences in bulk salinity, the diverse mineralogical assemblage within a volume of ice will uniquely affect its inherent characteristics. Different salt minerals affect ice properties (e.g., viscosity, eutectic temperature, liquid fraction as a function of temperature and composition, density, ice strength, etc.) in various ways (*6*, *87*). Moreover, in the often-used analogy between planetary ice shells and the terrestrial mantle-lithosphere system, even minor variations in petrology can introduce variations in material properties, melt fractions, and geodynamical processes (*12*, *13*, *90*, *91*). With diverse pathways for ice formation on ocean worlds (e.g., slow freezing from dilute oceans at the ice shell–ocean interface, fast freezing of intruded ocean water into shallow regions of the ice shell, and the solidification of cryoconcentrated brine in perched water lenses) (*1*, *2*), constraining the dynamics and extent of ion fractionation under an array of thermochemical conditions will be essential in characterizing their effects on ice shell geophysical processes and the relationship between ice geochemical/geomorphological signatures (e.g., distinct mineralogical assemblages) and the interior environmental conditions that generate them. With ion fractionation being driven by both precipitation and diffusion processes, we expect both fast- and slow-freezing environments in planetary ice shells to be affected by fractionation dynamics, with precipitation-driven fractionation dominating fast-freezing regimes (e.g., intrusion of brines into shallow regions of ice shells and extrusion of brines onto ice shell surfaces) and diffusion-driven fractionation likely dominating in slower-freezing regimes (e.g., modern ice-ocean interfaces) where interstitial brines may struggle to reach saturation limits before diffusing. While shallow thermal gradients and ice growth rates representative of deep ice-ocean interfaces are difficult to recreate in the laboratory, the observation of increasingly efficient diffusion-driven fractionation with depth in several of our laboratory-grown ice columns suggests that this fractionation mechanism could be exceptionally efficient at slow-growing interfaces, provided they remain above the critical growth velocity for maintaining a cellular interface structure (*92*, *93*).

The depletion or accumulation of certain ions and/or the formation of solid salt species also has implications for the habitability and biosignature preservation potential of planetary ice-brine systems. The ionic composition of a brine governs several properties that control its ability to support and sustain biology and biological processes. These include water activity (which is directly linked to freezing point depression), ionic concentration, and chaotropicity/kosmotropicity (*9*–*11*, *94*–*96*). Accordingly, the distribution and geochemical evolution of brines within planetary ices will govern their spatiotemporal habitability [e.g., (*30*)]. The potential for ion fractionation to enhance and deplete relative ion concentrations in intra-ice and sub-ice brines will thereby complicate how we understand the role freezing, salt rejection, and cryoconcentration processes play in governing environmental habitability, as the physics associated with ion fractionation processes could drive the concentration of either biologically beneficial or detrimental chemical species. For example, if kosmotropic sulfate species are preferentially entrained in forming ices, the residual underlying brine could become increasingly chaotropic due to the accumulation of high-solubility chloride species. In addition, the speciation of solid salts accumulated due to precipitation reactions has implications for the long-term preservation of biology and biosignatures. Salt hydrates have exceptional biological preservation potential, providing protection from natural degradation processes and having more resilience to surface exposure than ice as they are much less susceptible to sublimation/dehydration (*97*–*99*). Simultaneously, however, interactions between near-surface radiation and salts can amplify the degradation of organic materials, and these effects vary between salt species (*100*, *101*). Moreover, in many salt-rich systems, there is active biological aggregation associated with hydrated salts, as they provide localized chemical gradients and a potential source of free water that can be exploited by organisms (*102*–*104*). In ice-brine systems, the same cryoconcentration process that drives salt rejection from forming ice crystals may also concentrate any organics and organisms in both interstitial and underlying brines (*105*), colocating biological material in high-salinity regions prone to salt hydrate formation. However, the preservation capabilities of salt hydrates differ, with variability in their susceptibility to environmental degradation processes [e.g., (*97*–*99*)]. As such, characterizing the speciation of precipitates will play a critical role in assessing the long-term biosignature preservation potential of hydrate-bearing planetary ices, a key consideration in our search for life on ocean worlds (*106*, *107*).

While the implications of ion fractionation for planetary ice shells are profound, both geophysically and astrobiologically, investigations of ion fractionation in saline ices are sparse, and the field is ripe for exploration. With prior studies limited to natural ice samples [e.g., (*25*, *27*, *38*)], only one of which explored ice formed from a nonmarine environment (*39*), additional laboratory investigations akin to those performed in the current study are needed to continue constraining the geochemically diverse array of ices that can be produced from putative planetary ocean chemistries. This includes the exploration of additional brine compositions, brine concentrations, driving thermal gradients, and freezing geometries [e.g., (*2*)] and constraining the microstructural characteristics and dynamics that control brine and ion mobility within saline ice systems [e.g., (*78*, *93*, *108*)]. In addition to providing empirical constraints on the dynamics and potential extent of ion fractionation processes in planetary ices, these laboratory investigations will establish a critical benchmark dataset with which to validate numerical models of planetary ice-brine systems and contextualize future spacecraft (or lander) observations. Currently, there are no models of saline ices (terrestrial or planetary) that include the physics associated with ion fractionation (i.e., multispecies diffusion and salt hydrate precipitation). Contemporary state-of-the-art reactive transport models of sea ice and planetary ice shells treat salinity as a state variable that includes the cumulative effect of all ion species on freezing point depression and brine density, with a single molecular diffusivity and a generalized saturation concentration/temperature where the formation of a eutectic solid emulates the precipitation of solid salt hydrates (*15*, *109*). Conversely, there exist studies where the geochemical evolution and precipitated mineralogical assemblages within a solidifying liquid water lens perched in a planetary ice shell have been simulated (*20*, *22*, *33*); however, these models do not consider the entrainment of salt in the newly formed ice, negating the possibility of ion fractionation, and are thus equilibrium geochemical models for the initial brine composition as water is removed from the system via ice formation. Future models of planetary ices that seek to include the dynamics of ion fractionation will need to integrate the physics of multiphase reactive transport with the low-temperature geochemical capabilities of a model like PHREEQC (*68*). While the iteration between the two model types will increase the complexity and computational expense of such a comprehensive model, the ability to forecast the detailed spatial and temporal geochemical evolution of planetary ice-brine systems is essential for our study of ice-ocean worlds across the solar system and beyond.

### Summary

Using top-down ice growth experiments, we have demonstrated that ion fractionation in planetary analog ices is a common phenomenon that occurs across diverse brine composition, brine concentration, and ice growth rate regimes. Combining our empirical measurements of ice ionic composition with geochemical equilibrium modeling, we have shown that positive ionic fractionation signals are consistent with the precipitation and retention of solid salt hydrates in the ice column and the retention of slowly diffusing ions, while negative ionic fractionation signals are consistent with the amplified diffusivities of certain ions in multispecies solutions and their subsequent relative depletion in the ice column. We have highlighted the significant variability we see in our experimental ion fractionation values (−40 to +77%) and discussed how these variations are likely driven by environmental factors (e.g., brine composition/concentration and local thermal gradient) that govern the multiphase dynamics of ice-ocean/brine interfaces. Given the breadth of thermochemical conditions experienced by putative ice-brine environments across the solar system, we suggest that ion fractionation is likely a widespread mechanism in planetary ices that must be further constrained and integrated into future models of ice-ocean worlds if we are to understand the geophysics and habitability of these high-priority targets. Specifically, controlled experiments are needed to (i) robustly and quantitatively derive functional relationships between ice geochemistry and parent brine composition/concentration and ice formation rate and (ii) identify unique ice fractionation and mineralogical signatures associated with parent brine properties and formation histories (e.g., lack of negative K^+^ fractionation and associated picromerite precipitation as a potential fingerprint of fast-freezing, high-salinity, MgSO_4_-rich brines like those of the Europa 3 experiment).

With the potential to generate ices that are chemically and mineralogically diverse, ion fractionation provides a physical mechanism—one that is understudied and deserves more attention—for introducing geochemical and material property heterogeneities into ice shells that could drive observed geophysical and geomorphological processes (*1*, *2*, *15*). Meanwhile, although planetary ice shells may contain information about their thermochemical formation environments, the processes associated with ion fractionation are distinctly capable of producing planetary ices with geochemical compositions that are unrepresentative of their parent fluid compositions. Moreover, the speciation and phase distribution of ions within ice-brine systems have substantial implications for the habitability of interstitial brines and the long-term preservation of biosignatures within solid salt hydrate assemblages. Ion fractionation offers a lens for the reanalysis of existing observational measurements and reinterpretation of ice-ocean world geophysics, geochemistry, and astrobiology.
