# Supplementary material for: As above, not so below: Ion fractionation in planetary analog ices
Source: Sci Adv. 2026 Apr 8;12(15):eady6763. doi: 10.1126/sciadv.ady6763 (PMC13060609; doi:10.1126/sciadv.ady6763)
Supplement: Supplementary file 1 — Supplementary Text Figs. S1 to S4 Legend for data S1 [file sciadv.ady6763_sm.pdf]

Science Advances
AAAS

# Supplementary Materials for

## As above, not so below: Ion fractionation in planetary analog ices

Jacob J. Buffo *et al.*

Corresponding author: Jacob J. Buffo, jacob.j.buffo@dartmouth.edu

*Sci. Adv.* **12**, eady6763 (2026)
DOI: 10.1126/sciadv.ady6763

**The PDF file includes:**

Supplementary Text
Figs. S1 to S4
Legend for data S1

**Other Supplementary Material for this manuscript includes the following:**

Data S1

**Supplementary Text**

S1: Cold Plate and Basal Brine Temperature Evolution

*In situ* temperature evolution of the overlying cold plate and underlying basal brine was monitored throughout all the experiments using Onset HOBO 64K Pendant dataloggers. Acquiring temperature readings every 5 minutes, this dataset provides a comprehensive understanding of the thermal environment during ice column growth, allows us to identify any operational temperature anomalies in the glycol chiller or cold room, and provides the plate-ice interface and ice-brine interface temperatures used to establish the conductive linear temperature profiles within the ice columns (e.g., Figure 3). An example of the plate and brine temperature evolution during the growth of the Seawater 2 sample can be seen in Figure S1. Experimental phases are clearly visible in the temperature plots; equilibration in the 2°C cold room prior to chiller activation and ice growth, ice growth (occurring between the vertical grey dashed lines), and temperature sensor extraction at the end of the experiments. Additional handheld probe thermometer readings of the cold plate temperature were taken at discrete intervals throughout the experiments to ensure consistent and accurate readings. All probe thermometer readings varied by less than a 1°C from the Onset HOBO 64K Pendant loggers.

There are a few features to note in the temperature data. First, cold plate temperatures do not reach the -20°C that the glycol chiller bath is set at. We attribute this to conductive heat loss from the glycol as it is circulating through the tubing connecting the chiller to the cold plate. The cold plate temperature decreases throughout the experiment. We attribute this to reduced heat loss as the tubing and cold plate accumulate frost throughout the experiments (e.g., Figure 1) and better insulate the glycol. Neither of these items are detrimental to our investigation goals/results as we are not concerned with a stable or specific magnitude of driving undercooling, but merely the ability to monitor its value as the experiment evolves. Second, small oscillations seen in the plate temperature evolution are associated with the thermal regulation cycling of the chiller bath temperature. Third, a brief power outage (< 4 hours) the morning of August 31$^{st}$, 2022 (spike in plate temperature – Figure S1) was detected, but as the plate temperature did not approach the metling point of the ice and the tank was well insulated we expect this brief anomaly did not measurably impact the growth or properties of the ice column. Another, more significant/longer power outage resulted in the loss of an ice column and that data was not included in this work. Fourth, cryoconcentration and freezing point depression in the underlying brine can be observed as the gradual decrease in the underlying brine temperatures recorded by the *in situ* logger as the experiment progresses (black line of Figure S1).

S2: Conductive Profiles in Laboratory Grown Ices

To validate the assumption that our fabricated ices possess linear conductive temperature profiles we conducted one experiment in which a vertical array of evenly spaced temperature sensors (Figure S2) was frozen into the growing ice. The resulting temperature evolution of the sensors can be seen in Figure S2 alongside the final thermal profile of the three sensors that were frozen into the ice at the time of its extraction. The thermal profile is strikingly linear, confirming the existence of a conductive profile in growing saline ices. The presence of the temperature sensors promoted a small amount of uneven ice growth in the tank, with slightly amplified ice thickness around the sensor array. The sensor array likely acted as a nucleation point for ice crystals as well as a conductive heat sink. Given the confirmed linearity of the ice thermal profiles and the desire to reduce any heterogeneities in ice growth, we excluded the temperature sensor array in all experimental runs used for geochemical analysis in the main manuscript text and

leverage the observed cold plate and underlying brine temperatures to linearly interpolate the final ice temperature profiles (Figure 3).

S3: Cryoconcentration of Underlying Brine and Subaqueous Salt Precipitation

During the extraction of the thickest ice column (Europa 2) there was a noticeable solid salt layer deposited at the base of the tank (Figure S3). Before experiments are begun, we ensure complete dissolution of all added salt species and the initial brine compositions are designed such that all minerals are below their saturation limits at 0°C. As such, the presence of this salt layer indicates that it formed due to the cryoconcentration and precipitation of salts in the residual underlying fluid as the overlying ice column formed. Preliminary Raman spectral analysis of this salt layer (Figure S4) suggests it is heavily dominated by gypsum, with potentially a small amount of a $MgSO_4$ salt (likely meridianiite given the brine temperature) mixed in. The dominance of gypsum in this salt layer is consistent with the strong negative $Ca^{2+}$ fractionation signal seen in the final underlying brine sample of this experiment, as precipitation is causing the brine to become further deplete in $Ca^{2+}$. Given the much larger concentration of $SO_4^{2-}$ any variations in fractionation cause by the sequestration of $SO_4^{2-}$ in the salt phase may not be as apparent. Such cryoconcentration and precipitation dynamics have direct implications for analogous processes thought to occur in isolated hydrological features within planetary ice shells such as sills and perched lenses [2, 7, 89].

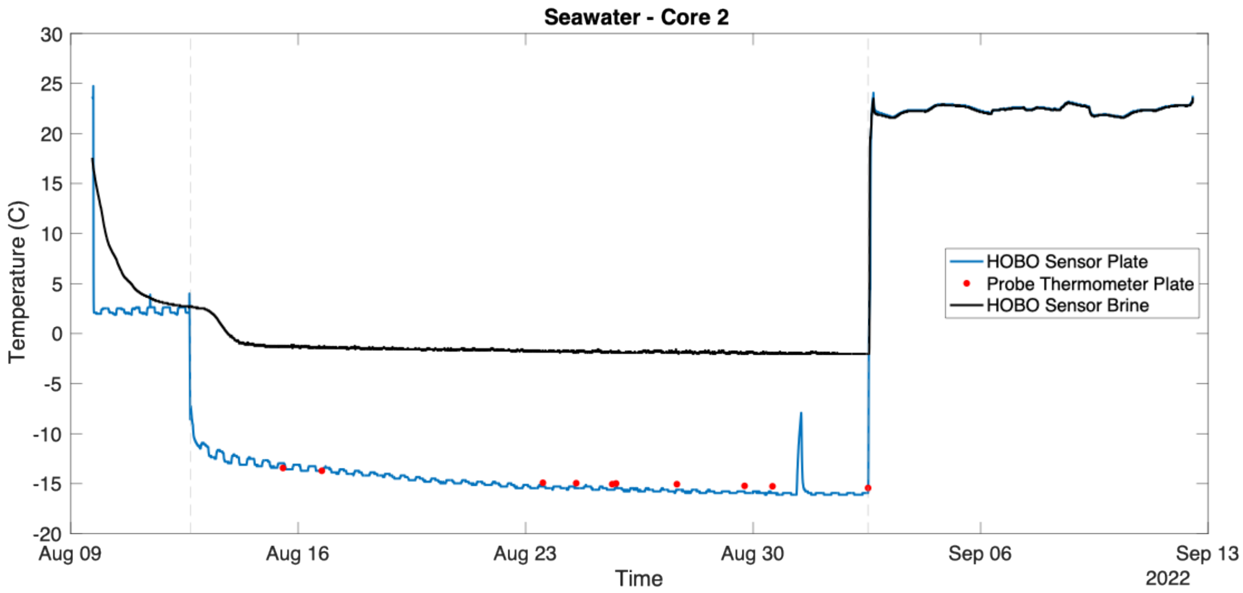


**Figure S1 – The thermal environment surrounding a laboratory-grown planetary analog ice column (specifically, Seawater 2).** Temperature loggers record the cold plate (black line) and underlying basal brine (blue line) temperatures during an experiment. Vertical dashed grey lines denote the onset (cold plate installation and activation) and completion of the experiment. Red dots denote additional cold plate temperature readings acquired using a handheld probe thermometer to validate the datalogger's accuracy.

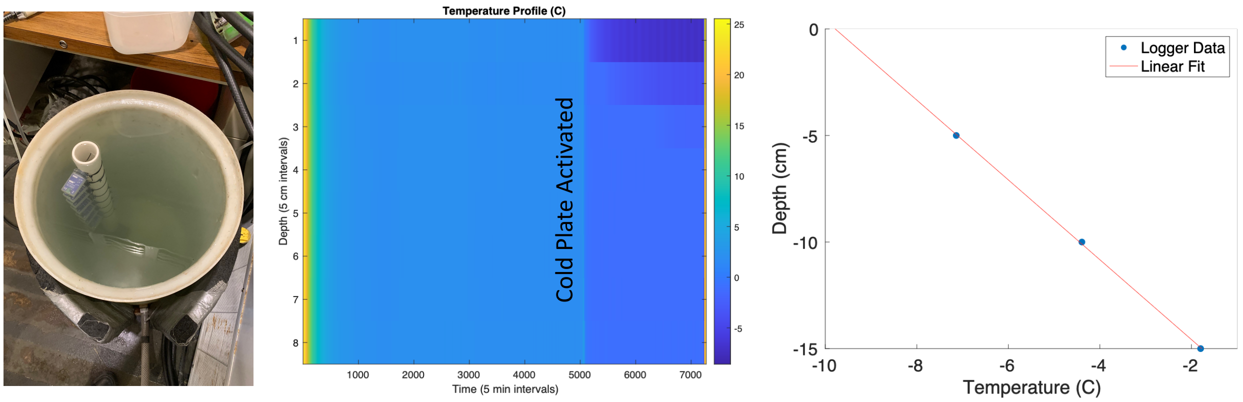


**Figure S2 – Acquisition of vertical temperature profiles in laboratory grown ices. Left)** The vertical temperature sensor array (5 cm spacing) deployed in a seawater solution prior to the emplacement and activation of the glycol chilled cold plate. **Center)** The temporal evolution of the temperature sensor array during a top-down growth experiment. The time prior to cold plate activation is used to equilibrate the brine to the ambient temperature of the cold room (2°C). The upper temperature sensors (1-3) become encased in the advancing ice as the experiment progresses, indicated by the noticeable temperature drops, while sensors in the well-mixed underlying brine remain at the liquid's freezing point temperature. **Right)** The temperature profile of the top three temperature sensors immediately before the experiments was stopped and the ice/sensor array was extracted (blue dots). A linear best fit line can be seen in red, exhibiting exceptional agreement with the temperatures measured by the sensors.

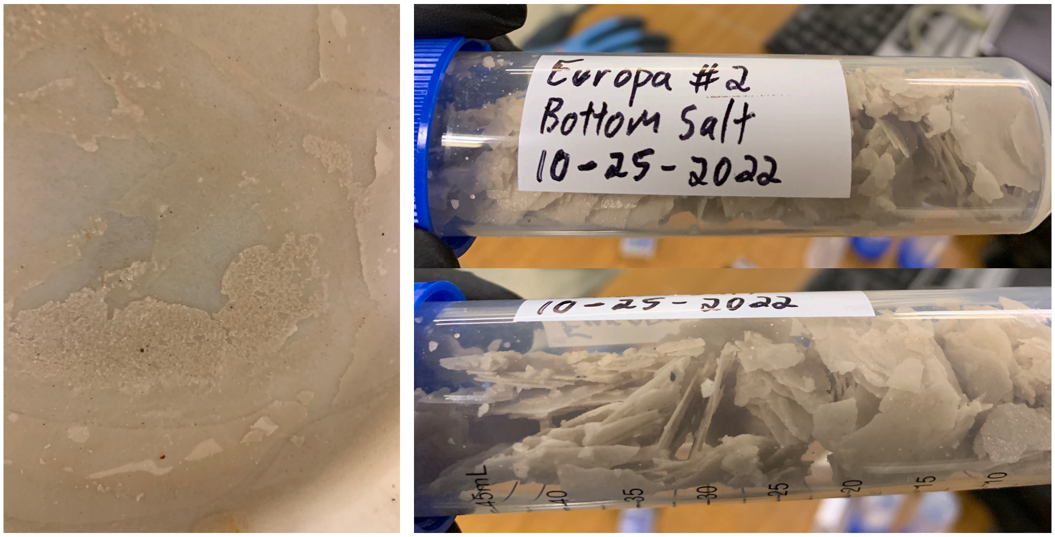


**Figure S3 – Subaqueous salt precipitates formed during the Europa 2 experiment.** The substantial thickness of this ice column (~50 cm, compared to a tank height of 65 cm) led to significant concentration, and ultimately saturation, of the underlying brine, causing a layer of salt to precipitate at the base of the tank. The initial brine concentrations (Table 1) are all tailored to be below their saturation point at 0°C, and we ensure all added salts are dissolved completely before the experiment is initiated, indicating these salts formed during the experiment as a result of cryoconcentration of the underlying brine.

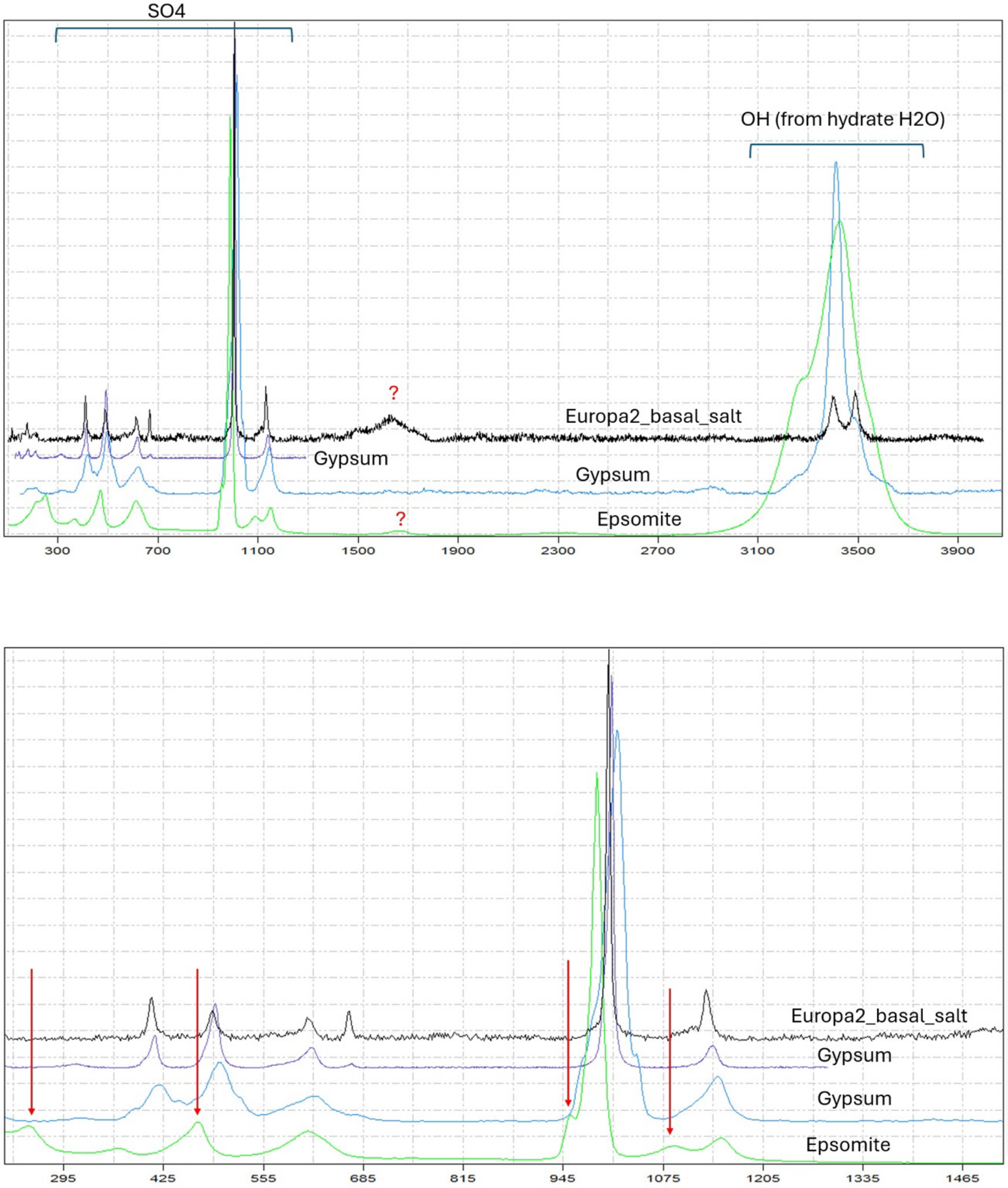


**Figure S4 – Preliminary Raman spectra of the subaqueous precipitated salt layer (Figure S3) and comparison to library spectra of potential salts. Top)** Full spectrum, highlighting potential contribution of a $MgSO_4$-bearing salt (red question marks). **Bottom)** Magnified view of the 230-1465 $cm^{-1}$ window of the spectra. Spectral signatures of the experiment-derived salt (black line) in this window are more consistent with those of gypsum (purple line) than they are with those of the $MgSO_4$-bearing epsomite (green line). Red arrows highlight features that would be expected in there was a significant abundance of a $MgSO_4$-bearing salt.

**Other Supplementary Materials for this manuscript include:**

**Data S1 – Ion concentration and fractionation data for all experiments.** The accompanying excel file 'Master_Chem' includes all of the experimental data presented in this manuscript.
